# Impaired Thymic Export and Apoptosis Contribute to Regulatory T-Cell Defects in Patients with Chronic Heart Failure

**DOI:** 10.1371/journal.pone.0024272

**Published:** 2011-09-15

**Authors:** Ting-Ting Tang, Zheng-Feng Zhu, Jun Wang, Wen-Cai Zhang, Xin Tu, Hong Xiao, Xin-Ling Du, Jia-Hong Xia, Nian-Guo Dong, Wei Su, Ni Xia, Xing-Xing Yan, Shao-Fang Nie, Juan Liu, Su-Feng Zhou, Rui Yao, Jiang-Jiao Xie, Harish Jevallee, Xiang Wang, Meng-Yang Liao, Guo-Ping Shi, Michael Fu, Yu-Hua Liao, Xiang Cheng

**Affiliations:** 1 Laboratory of Cardiovascular Immunology, Key Laboratory of Biological Targeted Therapy of the Ministry of Education, Institute of Cardiology, Union Hospital, Tongji Medical College of Huazhong University of Science and Technology, Wuhan, China; 2 Department of Cardiology, Yangzhou No. 1 People's Hospital, Yangzhou, China; 3 Key Laboratory of Molecular Biophysics of the Ministry of Education, Cardio-X Institute, College of Life Science and Technology and Center of Human Genome Research, Huazhong University of Science and Technology, Wuhan, China; 4 First Hospital of Wuhan, Wuhan, China; 5 Department of Cardiovascular Surgery, Union Hospital, Tongji Medical College of Huazhong University of Science and Technology, Wuhan, China; 6 Department of Medicine, Brigham and Women's Hospital and Harvard Medical School, Boston, Massachusetts, United States of America; 7 Department of Medicine, Sahlgrenska University Hospital, Gothenburg, Sweden; Institut Jacques Monod, France

## Abstract

**Objective:**

Animal studies suggest that regulatory T (T_reg_) cells play a beneficial role in ventricular remodeling and our previous data have demonstrated defects of T_reg_ cells in patients with chronic heart failure (CHF). However, the mechanisms behind T_reg-_cell defects remained unknown. We here sought to elucidate the mechanism of T_reg-_cell defects in CHF patients.

**Methods and Results:**

We performed flow cytometry analysis and demonstrated reduced numbers of peripheral blood CD4^+^CD25^+^FOXP3^+^CD45RO^−^CD45RA^+^ naïve T_reg_ (nT_reg_) cells and CD4^+^CD25^+^FOXP3^+^CD45RO^+^CD45RA^−^ memory T_reg_ (mT_reg_) cells in CHF patients as compared with non-CHF controls. Moreover, the nT_reg_/mT_reg_ ratio (*p<*0.01), CD4^+^CD25^+^FOXP3^+^CD45RO^−^ CD45RA^+^CD31^+^ recent thymic emigrant T_reg_ cell (RTE-T_reg_) frequency (*p<*0.01), and T-cell receptor excision circle levels in T_reg_ cells (*p<*0.01) were lower in CHF patients than in non-CHF controls. Combined annexin-V and 7-AAD staining showed that peripheral T_reg_ cells from CHF patients exhibited increased spontaneous apoptosis and were more prone to interleukin (IL)-2 deprivation- and CD95 ligand-mediated apoptosis than those from non-CHF individuals. Furthermore, analyses by both flow cytometry and real-time polymerase chain reaction showed that T_reg_-cell frequency in the mediastinal lymph nodes or Foxp3 expression in hearts of CHF patients was no higher than that of the non-CHF controls.

**Conclusion:**

Our data suggested that the T_reg_-cell defects of CHF patients were likely caused by decreased thymic output of nascent T_reg_ cells and increased susceptibility to apoptosis in the periphery.

## Introduction

Chronic heart failure (CHF) is regarded as a state of chronic inflammation with elevated T-cell activation and inflammatory cytokine production in the circulatory system [1], [2]. However, the pathogenic mechanisms responsible for this abnormal immune activation remain unknown. T_reg_ cells represent a unique lineage of T cells that play an essential role in the modulation of immune responses and the control of potentially harmful immune activations because of their immunoregulatory and immunosuppressive characteristics [3]. Among the several types of T_reg_ cells that have been defined, one particular subset that constitutively expresses CD4, CD25 and the transcription factor Foxp3 has received much attention. Alterations in CD4^+^CD25^+^Foxp3^+^ T_reg_-cell number or function is directly associated with the pathogenesis of several common human diseases, including acute coronary syndrome (ACS) [4], [5], multiple sclerosis [6], type 1 diabetes [7], and rheumatoid arthritis [8]. Adoptive transfer of purified T_reg_ cells suppresses immune injury and improves recovery in animal disease models [9]–[12].

Adverse ventricular remodeling occurs upon acute and chronic injury regardless of etiology, and it is related to poor prognosis of patients with heart failure [13]. There is compelling evidence that inflammatory mechanisms contribute to the process of adverse ventricular remodeling [14]. In animal models of heart failure, previous studies demonstrated that T_reg_ cells could be a target of heart failure therapeutics because CCR5-mediated T_reg_-cell recruitment in the infarcted heart [15] and adoptively transferred T_reg_ cells [16] provided protection from adverse cardiac remodeling by preventing expansion of inflammation and fibrosis after adoptive transfer. In a previous publication, we found that circulating T_reg_ cells were reduced and their function was altered in CHF patients, regardless of etiology, suggesting that the defects in T_reg_ cells are responsible for the aberrant chronic immune activation in CHF patients [17]. It is believed that the understanding of mechanisms underlying T_reg_-cell defects in CHF patients is of great significance, especially with respect to therapy through T_reg_-cell manipulation. In the present study, we attempt to explore the mechanisms that might account for the T_reg_-cell defects in CHF patients by studying T_reg_-cell production, survival, and tissue reallocation in these patients.

## Results

### 1. Reduced nT_reg_-, mT_reg_- and RTE-T_reg_-cell frequency in CHF patients

**Figure 1:**
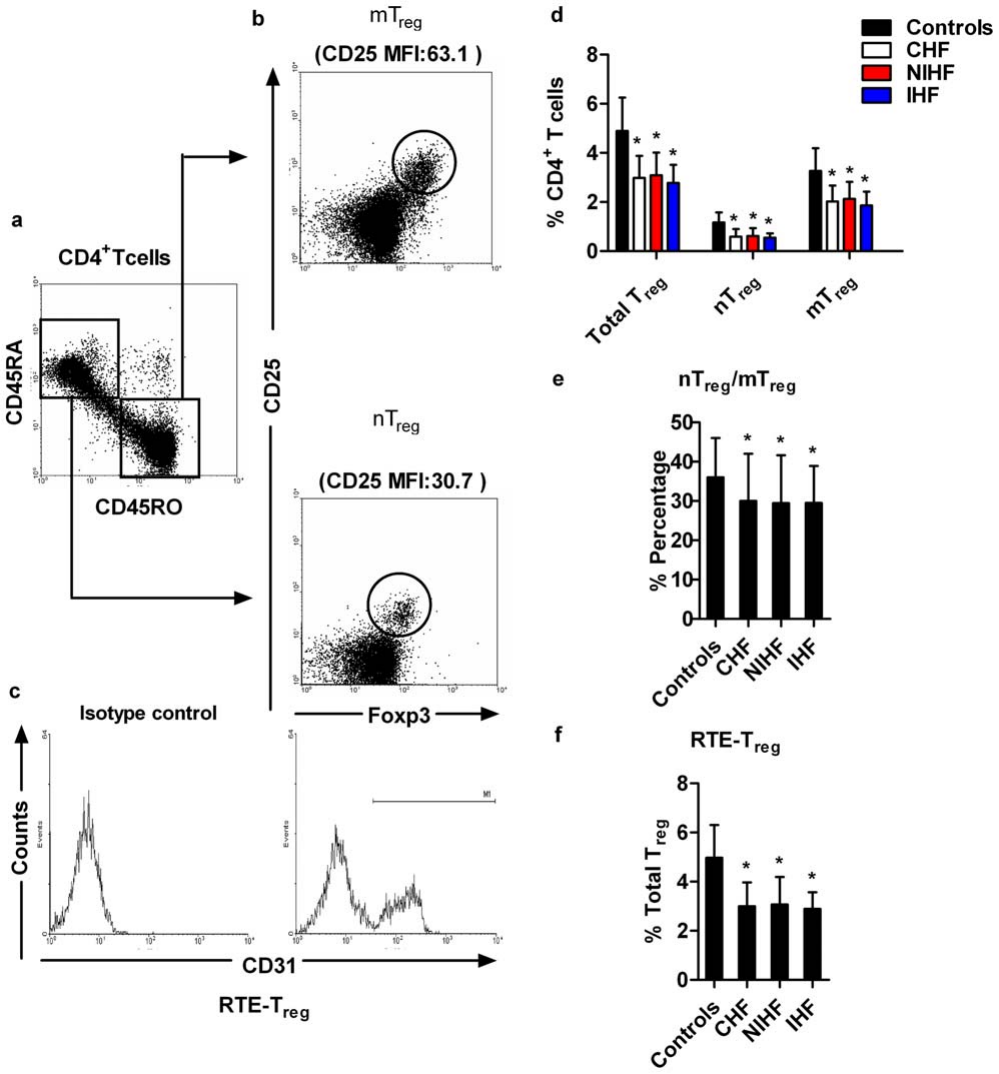
Frequencies of the regulatory T (T_reg_)-cell subset in CHF patients and non-CHF controls. PBMCs of CHF patients (n = 52, 32 NIHF and 20 IHF) and non-CHF controls (n = 43) were included, and a 6-color flow cytometric analysis using mAbs specific for CD4, CD25, CD45RA, CD45RO, CD31 and Foxp3 was performed. a**.** Representative FACS images from a non-CHF control. Dot plots show CD45RA and CD45RO expression on gated CD4^+^ T cells. Naïve and memory CD4^+^ T cells were defined as CD45RA^+^CD45RO^−^ (R1) and CD45RA^−^CD45RO^+^ (R2), respectively. b. A small subpopulation of memory T_reg_ (mT_reg_) (upper panel) and naïve T_reg_ (nT_reg_) (lower panel) cells expressing both CD25 and Foxp3 was detectable. c. Histograms show the expression of CD31 on nT_reg_ cells. Recent thymic export-T_reg_ (RTE-T_reg_) cells were identified as CD4^+^CD25^+^Foxp3^+^CD45RA^+^CD45RO^−^CD31^+^ cells. d. Frequencies of total T_reg_, nT_reg_, and mT_reg_ cells in different patient populations were determined as percentages of total CD4^+^ T cells. e. The ratio of nT_reg_ to mT_reg_ cells in different subject populations. f. RTE-T_reg_ cell frequency in different subject populations was presented as a percentage of total T_reg_ cells. *p<0.05 vs. non-CHF controls.

To determine the number of total T_reg_ cells and T_reg_ subsets, PBMCs were obtained from 52 CHF patients and 43 age-matched non-CHF controls followed by 6-color flow cytometric analysis. Basic clinical characteristics of the study population are summarized in Table 1. Within the naïve CD4^+^CD45RA^+^CD45RO^−^ (R1 in Figure 1A) or memory CD4^+^CD45RA^−^CD45RO^+^ (R2 in Figure 1A) T cells, a small subpopulation of cells with high expression of both CD25 and Foxp3 could be readily detected. mT_reg_ cells were characterized as CD4^+^CD25^+^Foxp3^+^ CD45RA^−^ CD45 RO^+^ cells (R3 in Figure 1B, upper panel) and nT_reg_ cells were characterized as CD4^+^CD25^+^Foxp3^+^CD45RA^+^CD45RO^−^ cells (R4 in Figure 1B, lower panel). nT_reg_ cells exhibited a lower expression of CD25 as compared with mT_reg_ cells (mean fluorescent intensity, MFI: nT_reg_ *vs.* mT_reg_: 24.1±5.4 *vs.* 54.6±8.7, *p<*0.01). RTE-T_reg_ cells were identified as CD31 co-expressing nT_reg_ cells (Figure 1C). The proportion of T_reg_ cells in total CD4^+^ T cells was significantly decreased in CHF patients when compared with non-CHF subjects (Figure 1D). The percentages of nT_reg_ and mT_reg_ cells within CD4^+^ T cells were also significantly lower in CHF patients than in age-matched non-CHF subjects (non-CHF *vs.* CHF patients: nT_reg_: 1.17±0.41% *vs.* 0.59±0.31%, *p<*0.01; mT_reg_: 3.27±0.92% *vs.* 2.02±0.65%, *p<*0.01; Figure 1D). CHF patients showed a significantly lower nT_reg_/mT_reg_ ratio (non-CHF *vs.* CHF patients: 36±10% *vs.* 30±12%, *p<*0.05; Figure 1E). Furthermore, we observed that proportions of RTE-T_reg_ cells in the total T_reg_-cell population in CHF patients were significantly reduced when compared to age-matched, non-CHF controls, suggesting that thymic production of T_reg_ cells was impaired in CHF patients (non-CHF *vs.* CHF: 4.97±.34% *vs.* 3.00±0.97%, *p<*0.01; Figure 1F). However, no difference in total T_reg_, nT_reg_, mT_reg_, and RTE-T_reg_ cells between IHF or NIHF patients was observed (Figure 1D–F). Similar results were obtained when we compared the absolute numbers of total T_reg_, nT_reg_, mT_reg_, and RTE-T_reg_ cells between CHF patients and non-CHF controls (Table 2).

**Table 1:** Clinical characteristics of study population.

	CHF patients (n = 52)	NIHF patients (n = 32)	IHF patients (n = 20)	Non-CHF controls(n = 43)
Age (year)	44±13	40±14	51±8	42±12
Gender (Male/Female)	31/21	17/15	14/6	28/15
NYHA (II/III/IV)	25/21/6	12/16/4	13/5/2	—
LVEF (%)	35.38±6.24	35.06±6.41	35.9±6.09	—
LVEDD (cm)	6.11±0.47	6.16±0.5	6.02±0.41	—
Hypertension (%)	37	16	70	0
NT-proBNP (pg/ml)	2955.52±1971.55	2756.33±1628.37	3176.8±2442.88	—
Medication (%)				
ACEI/ARBs	90	91	90	0
Antisterone	42	47	35	0
Digitalis	31	28	35	0
β-Blocker	87	88	85	0
Diuretics	73	50	80	0

Data is presented as mean±SD, or number or percentage of patients or healthy controls (HCs). NIHF: non-ischemic heart failure; IHF: ischemic heart failure; NYHA, New York Heart Association; LVEF, left ventricular ejection fraction; LVEDD, left ventricular end-diastolic dimension; NT-ProBNP, N-terminal Pro B-type natriuretic peptide; ACEI, angiotensin-converting enzyme inhibitor; ARB, angiotensin receptor blocker.

**Table 2:** Absolute number of T_reg_, nT_reg_, mT_reg_ and RTE-T_reg_ in the study population.

	CD4^+^ T cells (10^6^/L)	T_reg_ (10^6^/L)	nT_reg_ (10^6^/L)	mT_reg_ (10^6^/L)	RTE-T_reg_ (10^6^/L)
CHF patients (n = 52)	341.95±206.28	9.92±5.78*	1.92±1.24*	6.84±4.30*	0.31±0.24*
NIHF patients (n = 32)	346.55±205.81	10.52±6.26*	2.06±1.37*	7.38±4.81*	0.35±0.29*
IHF patients(n = 20)	334.60±212.16	8.96±4.92*	1.69±0.97*	5.97±3.27*	0.25±0.13*
Non-CHF controls (n = 43)	289.99±155.36	13.96±7.6	3.33±2.07	9.51±5.49	0.70±0.43

Data is presented as mean±SD. T_reg_, regulatory T cells; nT_reg_, naïve T_reg_; mT_reg_, memory T_reg_; RTE-T_reg_, recent thymic emigrant T_reg_. *p<0.05 vs. non-CHF controls.

Consistent with our previous report [17], we observed that total T_reg_ number was negatively correlated with NT-proBNP in CHF patients. Furthermore, the present study also found that NT-proBNP and nT_reg_ or mT_reg_ numbers were negatively correlated (Table 3).

**Table 3:** Correlation analysis between T_reg_ or its subset frequency and NT-proBNP in CHF patients.

	NT-proBNP		
	T_reg_	nT_reg_	mT_reg_
Coefficients	−0.589	−0.557	−0.446
*p* values	<0.01	<0.01	<0.01

NT-proBNP, N-terminal Pro B-type natriuretic peptide; T_reg_, regulatory T cells; nT_reg_, naïve T_reg_; mT_reg_, memory T_reg_.

### 2. Decreased intracellular TREC levels in T_reg_ cells from CHF patients

**Figure 2,:**
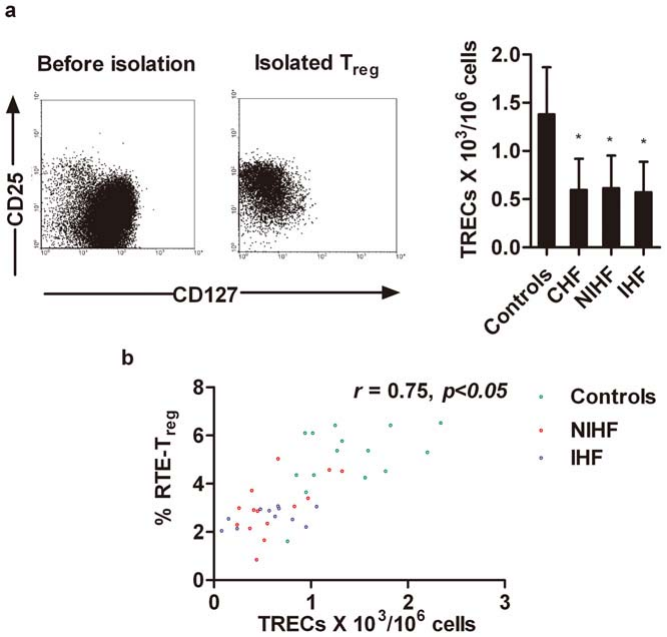
Analysis of intracellular T-cell receptor excision circle (TREC) levels in purified T_reg_ cells from CHF patients and non-CHF controls. a. CD4^+^CD25^+^CD127^low^ T_reg_ cells from CHF patients (n = 25, 14 NIHF and 11 IHF) and non-CHF controls (n = 15) were isolated by magnetic selection (left), and the TREC levels were determined by RT-PCR (right; *p<0.01) and compared to non-CHF controls. b. RTE-T_reg_ frequencies were plotted against TREC levels in purified T_reg_ cells from CHF patients and non-CHF controls (r = 0.75, *p*<0.001).

TREC is a marker for nascent thymic T cells [18]. We studied intracellular levels of TRECs in T_reg_ cells isolated from 25 CHF patients and 15 age-matched non-CHF subjects using quantitative real-time PCR. Flow cytometry was used to determine the purity of T_reg_ cells after cell sorting (Figure 2A, left panel). The TREC content in T_reg_ cells was significantly lower in CHF than that in non-CHF patients (non-CHF *vs.* CHF patients: 1.38±0.49×10^3^/10^6^ cells *vs.* 0.60±0.32×10^3^/10^6^ cells, *p*<0.01; Figure 2A, right panel). There was no significant difference in T_reg_-cell TREC content between IHF and NIHF patients. Spearman's correlation test revealed a positive association between T_reg_-cell TREC level and RTE-T_reg_ cell proportion in both CHF patients and non-CHF controls (r  =  0.75, *p*<0.001; Figure 2B).

### 3. Increased spontaneous and IL-2 deprivation/Fas-mediated apoptosis in T_reg_ cells from CHF patients

**Figure 3:**
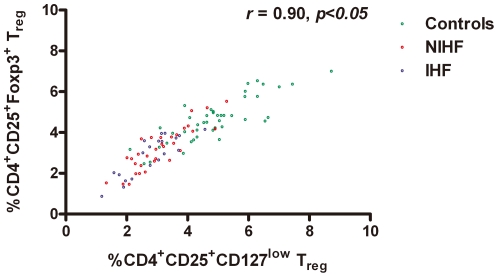
Correlation between CD4^+^CD25^+^ Foxp3^+^ T_reg_ cells and CD4^+^CD25^+^CD127^low^T_reg_ cells. Frequencies of CD4^+^CD25^+^Foxp3^+^ T_reg_ cells were plotted against CD4^+^CD25^+^CD127^low^T_reg_ cells in 47 CHF patients and 38 non-CHF controls (r = 0.91, *p<*0.001).

**Figure 4:**
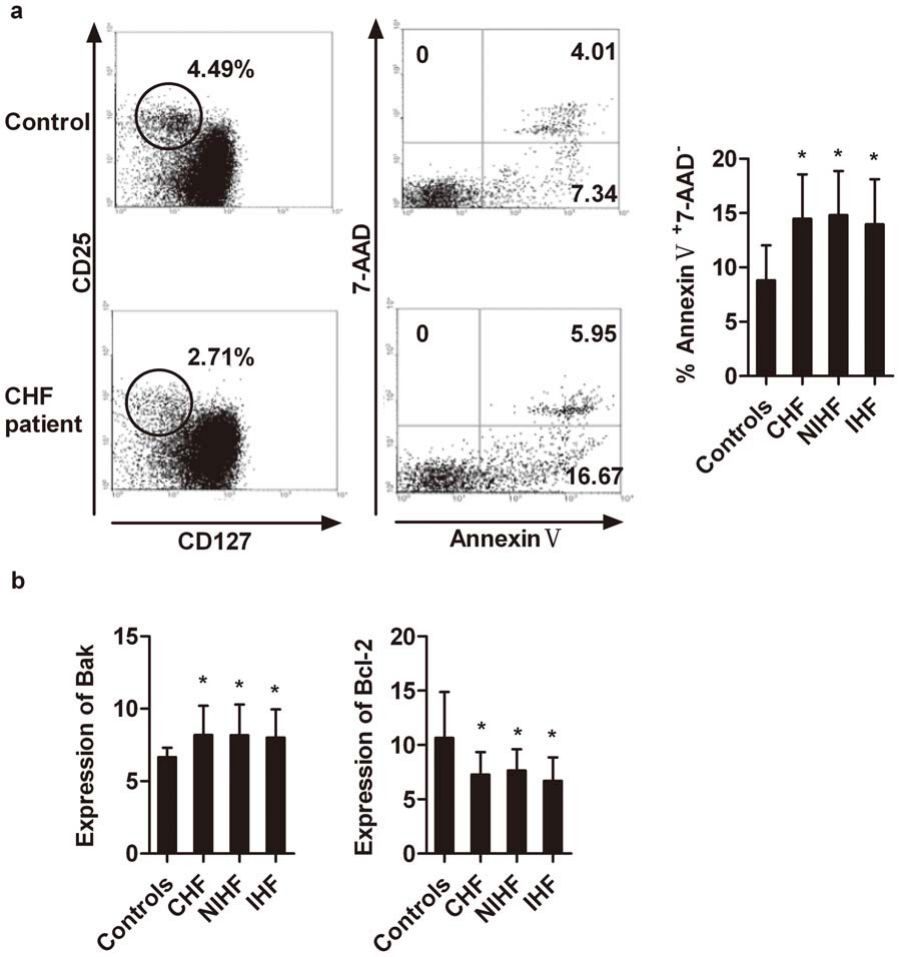
Spontaneous apoptosis of T_reg_ cells from CHF patients and non-CHF controls. PBMCs of 47 CHF patients and 38 non-CHF controls were stained with anti-CD4, anti-CD25, anti-CD127, annexin-V and 7-AAD and analyzed by flow cytometry. a**.** Representative FACS analyses from one non-CHF control and one CHF patient are shown. A small subpopulation of CD25^+^CD127^low/−^ cells were gated and identified as T_reg_ cells (left panels). The staining of annexin-V and 7-AAD was further analyzed on gated T_reg_ cells (middle), and apoptosis levels of the T_reg_ cells are calculated as percentage of annexin-V^+^7-AAD^−^ cells among 7-AAD^−^ cells (right; *p<0.01 *vs.* non-CHF controls). b**.** CD4^+^CD25^+^CD127^low/−^ T_reg_ cells from CHF patients (n = 25, 14 NIHF and 11 IHF) and non-CHF controls (n = 15) were isolated by magnetic selection (left), and the expression of both the anti-apoptotic gene Bcl-2 (top panel) and the pro-apoptotic gene Bak (bottom panel) was measured. *p<0.05 *vs.* non-CHF controls.

Increased apoptosis and decreased survival could be a mechanism of T_reg_-cell defects in CHF patients. Because of the fixation and permeabilization procedures used for detecting T_reg_ cells using Foxp3 antibodies by FACS, we detected apoptotic T_reg_ cells with antibodies against CD127, a newly identified T_reg_ surface marker that correlates well with Foxp3 [19]. In both CHF patients and non-CHF controls, CD4^+^CD25^+^Foxp3^+^ T_reg_ cells were correlated with CD4^+^CD25^+^CD127^low/−^ T_reg_ cells (r = 0.91, *p<*0.001, Figure 3). When we gated the CD4^+^CD25^+^CD127^low/−^ cells, we found that when this cell population was derived from CHF patients, irrespective of the etiology, there was a significantly higher percentage of apoptotic annexin V^+^7-AAD^−^ cells than when derived from non-CHF controls (non-CHF *vs.* CHF patients: 8.79±3.37% *vs.* 14.78±4.08%, *p*<0.01; Figure 4A).

Enhanced apoptosis often correlates with altered expression of apoptosis-associated genes. We compared the levels of anti-apoptotic gene Bcl-2 and pro-apoptotic gene Bak expression between CD4^+^CD25^+^CD127^low/−^ T_reg_ cells isolated from CHF patients and non-CHF controls. Significantly lower Bcl-2 expression (*p*<0.01) and higher Bak expression (*p*<0.01) were observed in T_reg_ cells from CHF patients when compared with those from non-CHF controls (Figure 4B).

**Figure 5:**
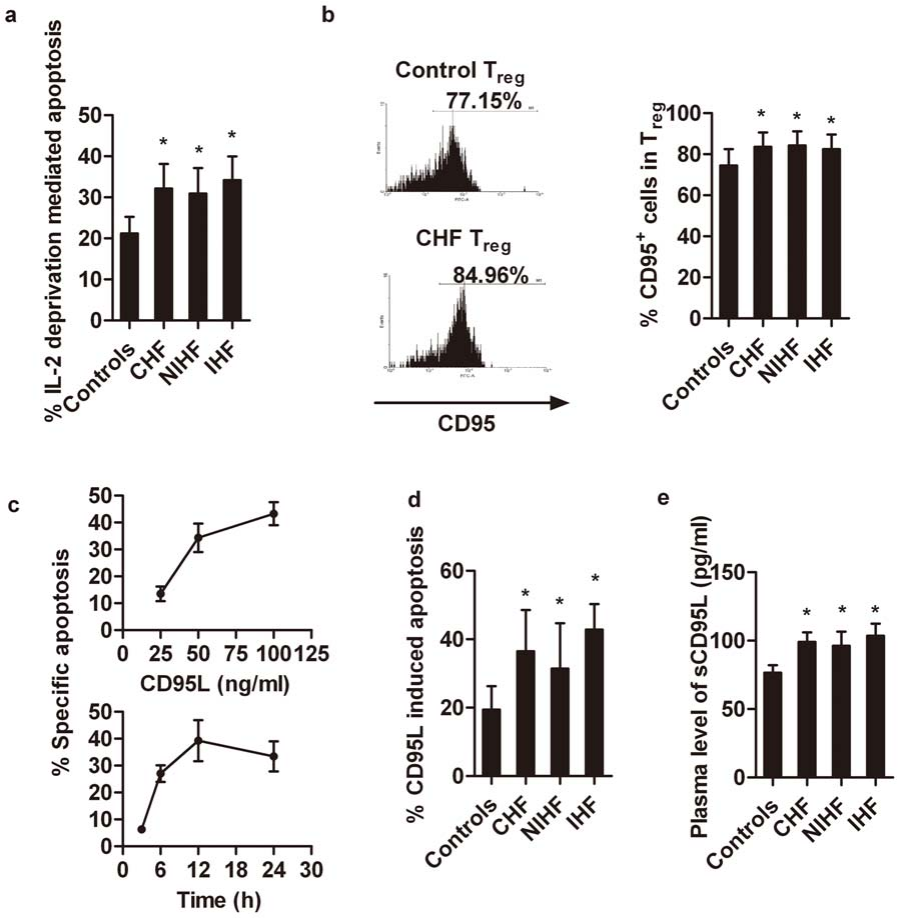
IL-2 deprivation and FasL-mediated T_reg_-cell apoptosis. PBMCs were stained with anti-CD4, anti-CD25, anti-CD127, and apoptosis was induced as described in Methods. a. IL-2 deprivation-mediated T_reg_-cell apoptosis between CHF patients (n = 47) and non-CHF controls (n = 38).b. CD95 expression on gated T_reg_ cells from CHF patients (n = 47) and non-CHF controls (n = 38). c. CD95L induced a dose-dependent apoptosis of T_reg_ cells from CHF patients after incubation with CD95L for 12 h (upper panel; data are means from three separate experiments). Apoptosis of T_reg_ cells from CHF patients in the presence of 100 ng/ml CD95L was plotted against time (lower panel; data are means from three separate experiments). d. FasL-induced apoptosis of T_reg_ cells from CHF patients and non-CHF controls (100 ng/ml FasL for 12 hrs). e. ELISA determination of plasma soluble FasL levels in 47 CHF patients and 38 non-CHF controls. *p<0.05 *vs.* non-CHF controls.

IL-2 is essential for the development, function and homeostasis of T_reg_ cells [20]. However, human T_reg_ cells do not produce this cytokine and therefore are susceptible to IL-2 deprivation, which leads to T_reg_-cell apoptosis [21]. T_reg_ cells from CHF patients and non-CHF controls might exhibit different susceptibilities to IL-2 deprivation. To test this hypothesis, we incubated T_reg_ cells from the different patient populations with anti-human IL-2 monoclonal antibodies for 3 days. T_reg_ cells from CHF patients were more sensitive to IL-2 deprivation-induced apoptosis when compared with T_reg_ cells from non-CHF subjects (non-CHF *vs.* CHF: 22.35±4.12% *vs.* 33.26±5.89%, *p*<0.01; Figure 5A).

T_reg_-cell apoptosis could also be induced by the interactions between death receptor CD95 with the CD95 ligand (CD95L) [22]. Human T_reg_ cells constitutively express these death receptors and are thus highly sensitive to CD95-CD95L-mediated apoptosis [23]. Increased apoptosis in T_reg_ cells from CHF patients suggests that T_reg_ cells from these patients express high levels of CD95 and/or are more sensitive to CD95L. To test this hypothesis, we compared the expression level of CD95 and sensitivity toward CD95L-triggered apoptosis in T_reg_ cells from CHF patients and non-CHF controls. CD95 expression on T_reg_ cells from CHF patients was significantly higher than on T_reg_ cells from non-CHF controls (non-CHF *vs.* CHF: 73.78±8.12% *vs.* 84.30±6.67%, *p<*0.01; Figure 5B). CD95L induced apoptosis of T_reg_ cells from CHF patients in a dose- and time-dependent manner (Figure 5C). CD95L initiated T_reg_-cell apoptosis in 3 hrs, but apoptosis reached a peak after 12 hrs of induction. When incubated with 100 ng/ml of CD95L for 12 hrs, T_reg_ cells prepared from CHF patients showed higher percentages of cells undergoing CD95L-induced apoptosis than in non-CHF subjects (non-CHF *vs.* CHF patients: 19.43±6.87% *vs.* 36.52±12.03%, *p<*0.01; Figure 5D). These observations could explain the increased T_reg_-cell apoptosis in CHF patients (Figure 4A). Furthermore, we detected significantly higher plasma levels of soluble CD95L in CHF patients than in non-CHF controls (non-CHF *vs.* CHF patients: 77.28±5.26% *vs.* 101.22±5.06%, *p*<0.01; Figure 5E). Among the CHF subgroups IHF and NIHF, we did not detect any differences in either IL-2 deprivation- or CD95-mediated T_reg_-cell apoptosis (Figure 5A/5D). Plasma CD95L levels were also similar between CHF, IHF, and NIHF patients (Figure 5E). Taken together, these findings suggest that T_reg_ cells from CHF patients were more prone to apoptosis and that IL-2 and CD95/CD95L might be involved in regulation of T_reg_-cell survival.

### 4. T_reg_ cells accumulate neither in mediastinal lymph nodes nor in failing hearts

**Figure 6:**
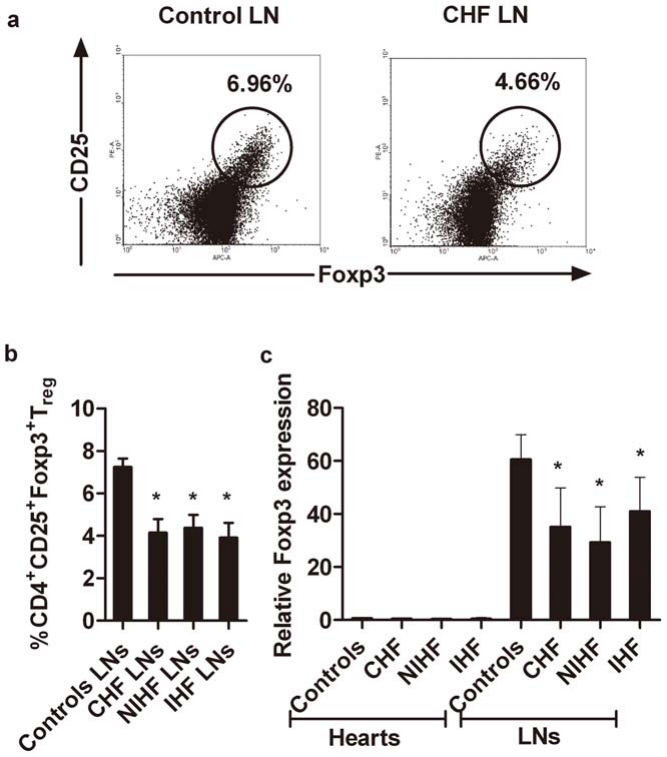
T_reg_ cells in mediastinal lymph nodes and hearts. a. Representative FACS dot plots showed the presence of CD4^+^CD25^+^Foxp3^+^ T_reg_ cells in the mediastinal lymph nodes. b. Percentages of CD4^+^CD25^+^Foxp3^+^ T_reg_ cells in the mediastinal lymph nodes were determined in six CHF patients (three with idiopathic cardiomyopathy and three with ischemic cardiomyopathy) and three controls without cardiomyopathy. c. Comparison of Foxp3 expression in the mediastinal lymph nodes and hearts of CHF and non-CHF controls. *p<0.05 *vs.* non-CHF controls.

One possible explanation for reduced T_reg_-cell number in CHF patients is the reallocation of these cells to the lymph nodes or disease-affected organs. We compared the proportion of CD4^+^CD25^+^Foxp3^+^ T_reg_ cells to total CD4^+^ T cells in the mediastinal lymph nodes from CHF patients and non-CHF controls. Mediastinal lymph node T_reg_ cells from CHF patients were significantly fewer than from non-CHF controls (Figure 6A/6B). Total lymphocyte Foxp3 mRNA levels were also significantly lower in CHF, IHF and NIHF patients than in non-CHF controls (Figure 6C). To examine whether T_reg_ cell accumulation in the heart was different between CHF and non-CHF controls, Foxp3 RT-PCR was performed on biopsied cardiac samples. No difference was found between failing hearts and hearts from donors, although Foxp3 levels were low in all tested heart samples (Figure 6C).

## Discussion

As the final common pathway of many cardiovascular diseases, CHF is a complex multi-step disorder and several mechanisms participate in its pathogenesis. There is compelling evidence that inflammation and autoimmune abnormalities play an important role in the progression of heart failure [1], [24], [25]. Various autoantibodies, which are directed against different cardiac antigens, such as cardiac myosin, cardiac troponin I, cardiolipin, beta_1_-adrenergic and M2 muscarinic receptors can be detected in the serum of patients with NIHF or IHF [26]–[29]. These autoantibodies can lead to cardiac injury, and they correlate with the deterioration of cardiac function. Other autoimmune abnormalities include infiltration of T cells in endomyocardial biopsies from patients with idiopathic dilated cardiomyopathy (DCM). Additionally, the transfer of peripheral blood lymphocytes from DCM patients to severe combined immunodeficiency (SCID) mice leads to ventricular remodeling [30]. In animal models, lymphocytes from rats with IHF can recognize and kill normal neonatal rat cardiac myocytes *in vitro* [31] and lead to autoimmune myocarditis *in vivo* after adoptive transfer [32].

T_reg_ cells play a key role in the control of inflammation and autoimmune responses, and altered T_reg_ cells predispose patients for uncontrolled immune activation or autoimmunity [3]. CHF patients were previously reported to have impaired T_reg_-cell number and function, but the precise mechanism behind this defect remains largely unknown [17]. In this study, we showed that reduced T_reg_ cell number and function in CHF patients might be explained by impaired T_reg_-cell thymic output and increased apoptosis of these cell populations.

Like other T cells, T_reg_ cells develop in the thymus [33]. A small fraction of T_reg_ cells with a naïve CD45RA^+^CD45RO^-^ surface profile (nT_reg_) has recently been detected in the circulation. However, this nT_reg_ subset declines with age, as does thymic output and other naïve T cells [34]. By contrast, the majority of circulating T_reg_ cells appear as a mature population with a memory CD45RA^−^CD45RO^+^ phenotype; these mT_reg_ cells are stable throughout the life span, and the levels of mT_reg_ cells increase during aging [35], [36]. nT_reg_ cells could represent the de novo generation of thymic lymphocytes, so the assessment of nT_reg_ cells is used to evaluate thymic T_reg_-cell production. In this study, we provided evidence that, in addition to decreased percentages of nT_reg_ and mT_reg_ cells, a shift from nT_reg_ cells toward mT_reg_ cells was evidenced by a reduced nT_reg_/mT_reg_ cell ratio in CHF patients. This result indicated the possibility that impaired thymic export contributes to T_reg_ cell defects in this patient population. However, nT_reg_ cells can proliferate after thymic output while retaining their naïve phenotype [37]. CD31 has been used as a direct marker of thymic output and enabled the discrimination of recent thymic emigrant (RTE) T_reg_ cells from peripherally expanded nT_reg_ cells [38]. Thus, the assessment of nT_reg_ cells co-expressing CD31 (RTE-T_reg_) is now used to evaluate the thymic output of T_reg_ cells. The significant reduction of peripheral RTE-T_reg_ cell content in CHF patients, when compared to the non-CHF controls, suggests a reduction of thymic T_reg_-cell output during the development of heart failure. An alternative approach to determine impaired T_reg_-cell thymic output in CHF patients was to assess intracellular concentration of TRECs in purified T_reg_ cells. TRECs are generated during the process of T-cell receptor rearrangement in T-cell differentiation and do not duplicate during mitosis. TRECs are diluted out during homeostatic or antigen-driven T-cell proliferation in the periphery [18]. Therefore, TRECs are enriched in the newly synthesized and exported T-cell pool. nT_reg_ cells, especially RTE-T_reg_ cells, have higher frequencies of TRECs as compared with mT_reg_ cells [38]. TREC content reduction in total T_reg_ cells from CHF patients further supported our hypothesis that the Treg-cell output in the thymus of a CHF patient is functionally altered. Hass *et al.* recently reported that T_reg_ cells from patients with and without multiple sclerosis showed different activities in suppressing T-effector cells. However, such differences disappeared after depleting the RTE-T_reg_ cells, indicating a crucial role of RTE-T_reg_ cells in the functional properties of the entire T_reg_ population [38]. Thus, impaired thymus export of T_reg_ cells could be associated not only with the number but also with the functional defect of T_reg_ cells in CHF patients. Over the course of multiple sclerosis, for example, patients appear capable of amplifying mT_reg_-cell subpopulations to compensate for impaired thymic production of T_reg_ cells [39]. In the case of CHF, in contrast, the homeostatic control of T_reg_ cells seems to be disturbed. Both nT_reg_ and mT_reg_ cells were reduced in CHF patients (Figure 1D).

The homeostasis of T_reg_ cells is maintained by a balance between T_reg_-cell generation and depletion. Apoptosis-induced alteration of T_reg_-cell levels has been associated with several diseases. For example, intrathyroidal CD4^+^CD25^+^ T_reg_ cells from patients with autoimmune thyroid diseases were prone to apoptosis, which led to a local T_reg_-cell reduction [40]. In contrast, patients with metastatic epithelial cancer demonstrated a significantly elevated proportion of peripheral T_reg_ cells, and these cells were protected from apoptosis [41]. Apoptosis not only reduces the number of T_reg_ cells, but also reduces their functions. By using T-effector cell suppression assays, T_reg_-cell apoptosis was closely associated with their capacity to inhibit T-effector cell proliferation [42]. In patients with type 1 diabetes, an increase in apoptosis was correlated with a decline in the suppressive potential of T_reg_ cells [43]. As suggested by these studies, high sensitivity to IL-2 deprivation or FasL-induced apoptosis may contribute in part to the defect of T_reg_ cells in CHF patients. T_reg_ cells from CHF patients were more susceptible to apoptosis following IL-2 deprivation. Upon antigen activation, T cells induce the expression of CD95, a member of the tumor necrosis factor receptor/nerve growth factor receptor superfamily that induces apoptosis by binding to CD95L and subsequently activating caspase [44]. In the present study, we demonstrated that T_reg_ cells in CHF patients had higher CD95 expression levels and were more sensitive to CD95/CD95L-mediated apoptosis than those in non-CHF subjects. Indeed, we also detected concurrent increases in serum soluble CD95L levels in CHF patients, consistent with prior observations [45]. These findings strongly suggest that the CD95/CD95L pathway is an important regulator of T_reg_-cell apoptosis in CHF patients.

After release from the thymus, T_reg_ cells circulate continuously from blood to lymphoid tissues. In disease conditions, the expression of chemokine receptors, such as CCR4 and CCR8, on T_reg_ cells allows their migration and recruitment to the site of inflammation [46]. In several human diseases, T_reg_ cells preferentially accumulate at lymphoid tissues or sites of affected organs [47], [48]. Therefore, it is possible that decreases in peripheral T_reg_ cells in CHF patients are caused by T_reg_-cell reallocation rather than an overall decrease. To investigate this possibility, we compared the T_reg_-cell numbers in the mediastinal lymph nodes or Foxp3 expression in cardiac biopsies between CHF patients and non-CHF controls. The results revealed that T_reg_-cell frequency in the mediastinal lymph nodes or Foxp3 expression in hearts of CHF patients was no higher than that of the non-CHF controls. However, this possibility could not be excluded due to the very small sample number. In addition to generation in the thymus, T_reg_ cells can also be converted from activated effector or memory CD4^+^CD25^−^ T cells in the periphery [49]. Peripherally converted T_reg_ cells and thymus-generated T_reg_ cells demonstrate a similar phenotype and suppressive functions. It is possible that such peripheral T-cell phenotype conversion was altered in CHF patients. This hypothesis merits further investigation.

TNF-α is central in the inflammatory cytokines response in CHF and play a role in the pathogenesis and clinical progression of the disease [50]. IL-10, an anti-inflammatory cytokine, may offer protection against TNF-α and an improvement in cardiac function in CHF has been associated with an increase in IL-10 [51] or a decrease in TNF-α/IL-10 ratio [52]. Our data indicated that T_reg_ frequency was negatively correlated with serum level of TNF-α or the TNF-α/IL-10 ratio (Figure S1). In both our previous study [17] and the present study, we observed that total T_reg_ number was significantly negatively correlated NT-proBNP which is considered as the most sensitive index of cardiac dysfunction in CHF patients. Based on these observations, we may speculate that T_reg_ cells provided protection for the failing heart and defects in T_reg_ cells is involved in the deterioration of cardiac function in CHF patients. However, the direct effect of T_reg_ cells on cardiac dysfunction still needs to be studied in animal model.

IL-10 and TGF- β1 have been identified as the main effector cytokines of T_reg_ cells [53]. We investigated the hypothesis that impaired T_reg_-cell function was associated with the decreased expression of these two cytokines. Disappointedly, we failed to observe a decrease in the expression of either IL-10 or TGF- β1 in CHF patients (Figure S2).

To conclude, our study revealed that both impaired export from the thymus and enhanced apoptosis can account for impaired T_reg_-cell number and function in CHF patients, offering a mechanistic explanation for the phenotypes and providing possible novel targets for CHF therapy through T_reg_-cell manipulation.

## Materials and Methods

### 1. Subjects

samples were collected from 52 CHF patients (31 men and 21 women, 44±13 years old) and 43 non-CHF controls (28 men and 15 women, 42±12 years old). Peripheral blood mononuclear cells (PBMCs) were prepared by Ficoll density gradient centrifugation (Sigma, USA). Plasma was obtained after centrifugation and stored at −80°C. CHF diagnoses were based on clinical history, physical examination, echocardiography, chest X-ray, electrocardiography and levels of N-terminal pro-brain natriuretic peptide (NT-proBNP), according to available guidelines pertaining to CHF. Patients were classified as having non-ischemic heart failure (NIHF) (n = 32, 17 men and 15 women) if they had no history of myocardial infarction and did not have significant coronary artery stenosis upon coronary angiography. Patients were considered to have ischemic heart failure (IHF) (n = 20, 14 men and 6 women) if the coronary angiography presented significant coronary artery disease (>50% stenosis in more than one major epicardial coronary artery) or the patients had a history of myocardial infarction or previous revascularization. Patients were excluded (1) if they were currently treated with anti-inflammatory drugs, such as non-steroidal anti-inflammatory drugs and steroids, (2) if they had collagen disease, thromboembolism, disseminated intravascular coagulation, advanced liver disease, renal failure, malignant disease, other inflammatory disease (such as septicemia, pneumonia), valvular heart disease, or atrial fibrillation, or (3) if they had pacemakers. Patients with higher serum cholesterol than the target values after risk stratification [54], who received statin therapy within 3 months, were also excluded. Mediastinal lymph nodes [55] and left ventricular biopsies were obtained from six CHF patients (three patients with dilated cardiomyopathy who underwent cardiac transplantation and three patients with coronary heart disease who underwent the combined bypass surgery and left ventricular aneurysm resection) and three controls (heart graft donors without cardiomyopathy who died in car accidents).

### 2. Ethics statement

The investigation conforms to the principles outlined in the Declaration of Helsinki. The trial was approved by the ethics committee of Tongji Medical College of Huazhong University of Science and Technology and patients and controls provided written informed consent.

### 3. Naïve T_reg_ (nT_reg_), memory T_reg_ (mT_reg_) and recent thymic emigrant-T_reg_ (RTE-T_reg_) cells in the circulation

A 6-color flow cytometry analysis was performed to determine levels of nT_reg_, mT_reg_ and RTE-T_reg_ in the circulation. PBMCs were stained with surface antibodies for APC/Cy7 anti-human CD4, PE anti-human CD25, FITC anti-human CD45RA, Percp/Cy5.5 anti-human CD45RO and PE/Cy7 anti-human CD31 (Biolegend) for 30 min at 4°C. After surface staining, cells were fixed, permeabilized, and stained with APC anti-human Foxp3, according to the manufacturer's instructions (eBioscience, USA). Antibody isotype controls were performed to ensure antibody specificity. Stained cells were analyzed by flow cytometry with FACS Aria (BD Biosciences, USA).

### 4. T_reg_−cell isolation

A two-step selection using a CD4^+^CD25^+^CD127^dim/−^ Regulatory T cell Isolation Kit (Miltenyi Biotec, Germany) was used to isolate T_reg_ cells according to the manufacturer's instructions. Briefly, non-CD4^+^ and CD127^high^ cells were magnetically labeled with a cocktail of biotin-conjugated antibodies and anti-biotin microbeads and subsequently depleted by negative selection. Pre-enriched CD4^+^ T cells were then labeled with anti-CD25 microbeads, and CD4^+^CD25^+^CD127^dim/−^ T_reg_ cells were isolated by positive selection. FACS was used to confirm the purity (>90%) of the isolated T_reg_ cells.

### 5. Quantification of T-cell receptor excision circles (TRECs)

The Wizard® Genomic DNA Purification Kit (Promega, USA) was used to extract genomic DNA (gDNA) from purified T_reg_ cells. Quantitative real-time PCR on an ABI Prism 7900 sequence detection system (Applied Biosystems, USA) was used to determine the number of TRECs. Primer pairs and probes were as follows:

TREC: F: 5′-aacagcctttgggacactatcg-3′, R: 5′-gctgaacttattgcaactcgtgag-3′, probe: 5′-6FAM-ccacatccctttcaaccatgctgacacctc-TAMAR-3′;

RAG2: F: 5′-gcaacatgggaaatggaactg-3′, R: 5′-ggtgtcaaattcatcatcaccatc-3′, probe: 5′-6FAM-cccctggatcttctgttgatgtttgactgtttgtga-TRAMRA-3′. Data were expressed as TRECs/10^6^ cells.

### 6. Apoptosis assays

Freshly isolated PBMCs were first stained with surface antibodies APC/Cy7 anti-human CD4, PE anti-human CD25, Percp/Cy5.5 anti-human CD95 (Fas) (Biolegend, USA) and Alexa Fluor® 647 anti-human CD127 (eBioscience, USA). Cells with the phenotype CD4^+^CD25^+^CD127^low^ were identified as T_reg_ cells. Apoptosis was measured using annexin V and 7-aminoactinomycin D (7-AAD) co-staining (Bender MedSystems, USA). The proportion of annexin V^+^7-AAD^−^ apoptotic cells in 7-AAD^−^ viable T_reg_ cells and the surface expression of CD95 on T_reg_ cells were analyzed using FACS Aria (BD Biosciences, USA).

For IL-2 deprivation-mediated apoptosis, cells were stimulated with 2 µg/ml plate-bound anti-CD3 (eBioscience, USA) and anti-human IL-2 monoclonal antibodies (Peprotech, 2 µg/ml) for 3 days. For Fas ligand (FasL)-induced apoptosis, cells were stimulated with gradient concentrations of soluble recombinant FasL (Peprotech, USA) in complete RPMI1640 containing IL-2 (100 IU/ml) for 12 hrs [56]. CD4^+^CD25^+^CD127^low/−^ T_reg_ cells were also gated for annexin V^+^7-AAD^–^ to determine apoptotic cell populations. Cell death was presented as (Percent of IL-2 deprivation- or FasL-mediated apoptosis - percent of apoptosis in the absence of anti-human IL-2 or FasL) / (100% - percent of cells in the absence of anti-human IL-2 or FasL)×100.

### 7. Soluble CD95 ligand (sCD95L) ELISA detection

Human FasL/TNFSF6 Quantikine ELISA Kit (R&D Systems, USA) was used to determine the plasma sCD95L levels. The minimal detectable concentration was 2.66 pg/ml, and intra-assay and inter-assay coefficients of variation were <10%. All samples were measured in duplicate.

### 8. T_reg_-cell detection in mediastinal lymph node

Mediastinal lymph nodes were minced and filtered through a cell strainer to create a single cell suspension preparation. Lymphocytes were isolated using Ficoll-Hypaque, stained with specific antibodies for CD4, CD25 and Foxp3, and subjected to FACS analysis. The number of T_reg_ cells in the lymph nodes was quantified by flow cytometry.

### 9. Real-time PCR

Total RNA was extracted using Trizol lysis buffer (Invitrogen, USA), and cDNA was prepared using the Revertra Ace® kit (Toyobo, Japan). Expression of target genes (Bcl-2 and Bak in purified T_reg_ and Foxp3 cells) in heart tissues and lymphocytes isolated from mediastinal lymph nodes was quantified using the SYBR Green Master Mix (Takara, Japan) on an ABI Prism 7900 Sequence Detection system (Applied Biosystems, USA). Primer pairs were as follows:

Bcl-2: F, 5**-**tacctgaaccggcacctg**-**3, R, 5**-**gccgtacagttccacaaagg-3;

Bak: F, 5-cctgccctctgcttctgag-3, R, 5-ctgctgatggcggtaaaaa-3;

Foxp3: F, 5′-gaaacagcacattcccagagttc-3′, R, 5′-atggcccagcggatgag-3′

GAPDH: F, 5′-ccacatcgctcagacaccat-3′, R, 5′-ggcaacaatatccactttaccagagt-3′

For each sample, the mRNA expression level was normalized to that of GAPDH. The mean of duplicate measurements was normalized and expressed as a ratio of target mRNA copies to GAPDH mRNA copies.

### 10. Quantification of transforming growth factor (TGF)-α and interleukin (IL)-10 expression in T_reg_ cells

PBMCs were cultured in RPMI1640 containing 10% FBS and stimulated with PMA (50ng/ml; Sigma-Aldrich, USA), ionomycin (1 µg/ml, Sigma-Aldrich, USA) and monensin (1 µΜ, eBioscience, USA) for 4 h. Cells were stained with surface antibodies for APC/Cy7 anti-human CD4, PE anti-human CD25 (Biolegend, USA), Alexa Fluor® 647 anti-human CD127 (ebioscience, USA) for 30 min at 4°C. After surface staining, cells were fixed, permeabilized, and stained with PE-cy7 anti-human TNF-α or PE-cy7 anti-human IL-10 (Biolegend, USA). Stained cells were analyzed by flow cytometry with FACS Aria (BD Biosciences, USA).

### 11. Tumor necrosis factor (TNF)-α and IL-10 ELISA detection

Commercial ELISA Kits (Invitrogen, USA) were used to determine the plasma TNF-α and IL-10 levels. The minimal detectable concentrations were 0.5 pg/ml and 0.78 pg/ml for TNF-α and IL-10 respectively. All samples were measured in duplicate.

### 12. Statistical analysis

Values are expressed as means ± standard deviation (SD) or percentage in the text and figures. For variables with normal distribution and homogeneity of variance, independent *t*-test or one-way analysis of variance (ANOVA) was used to test differences among two or more groups. For skewed variables, either non-parametric Kruskal-Wallis H test or Mann-Whitney *U* test were used for analyses. For the ranked data, Pearson's chi-square test or Fisher's exact test were used for the comparison between multiple groups. Spearman's correlation analysis was performed to determine correlation between the variables. In all cases, two-tailed, *p*<0.05 was considered significant.

## Supporting Information

Figure S1 **Correlation analysis between T_reg_ frequency and plasma levels of cytokines in CHF patients (n = 20).**
(TIF)

Figure S2 **Comparison of intracellular IL-10 and TGF-β1 in CD4^+^CD25^+^CD127^low^ T_reg_ between CHF patients (n = 10) and healthy controls (n = 10).**
(TIF)
