# Correction: Impaired Thymic Export and Apoptosis Contribute to Regulatory T-Cell Defects in Patients with Chronic Heart Failure

**DOI:** 10.1371/annotation/bc0b9ac0-b1d4-44ff-aa8c-cb27e199c1b1

**Published:** 2011-09-23

**Authors:** Ting-Ting Tang, Zheng-Feng Zhu, Jun Wang, Wen-Cai Zhang, Xin Tu, Hong Xiao, Xin-Ling Du, Jia-Hong Xia, Nian-Guo Dong, Wei Su, Ni Xia, Xin-Xin Yan, Shao-Fang Nie, Juan Liu, Su-Feng Zhou, Rui Yao, Jiang-Jiao Xie, Harish Jevallee, Xiang Wang, Meng-Yang Liao, Guo-Ping Shi, Michael Fu, Yu-Hua Liao, Xiang Cheng

The correct spelling of the 12th author's name is: Xin-Xin Yan 

