# Supplementary figures and images for: Impaired Thymic Export and Apoptosis Contribute to Regulatory T-Cell Defects in Patients with Chronic Heart Failure
Source: PLoS One. 2011 Sep 15;6(9):e24272. doi: 10.1371/journal.pone.0024272 (PMC3174174; doi:10.1371/journal.pone.0024272)

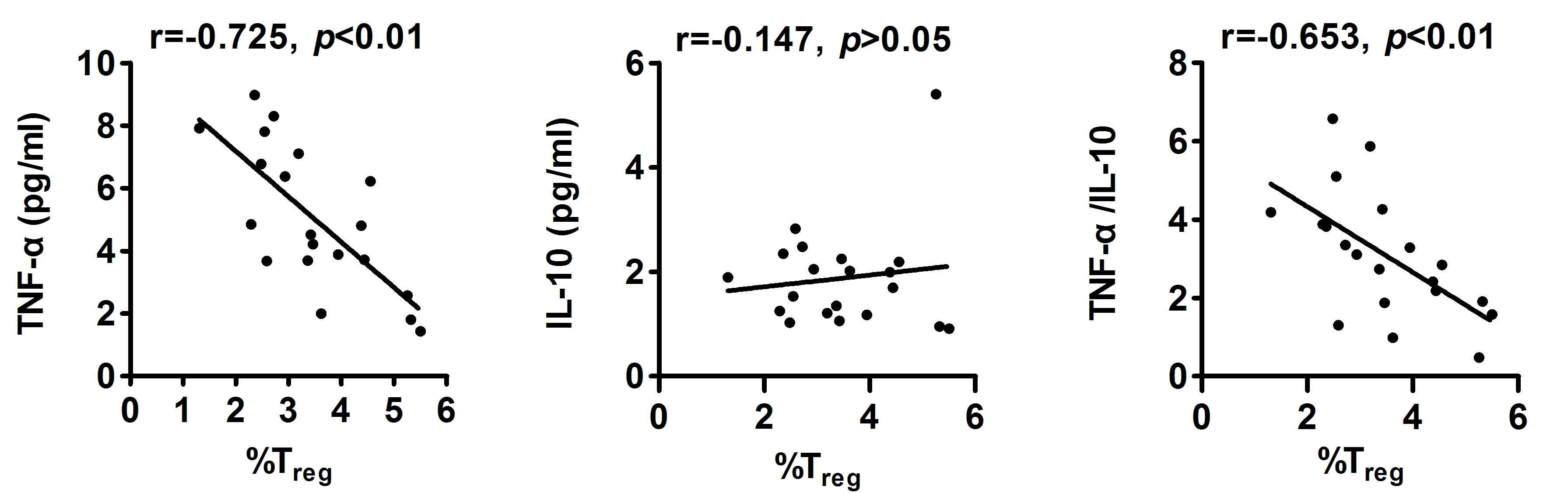

Supplement: Figure S1 — Correlation analysis between Treg frequency and plasma levels of cytokines in CHF patients (n = 20). (TIF) [file pone.0024272.s001.tif]

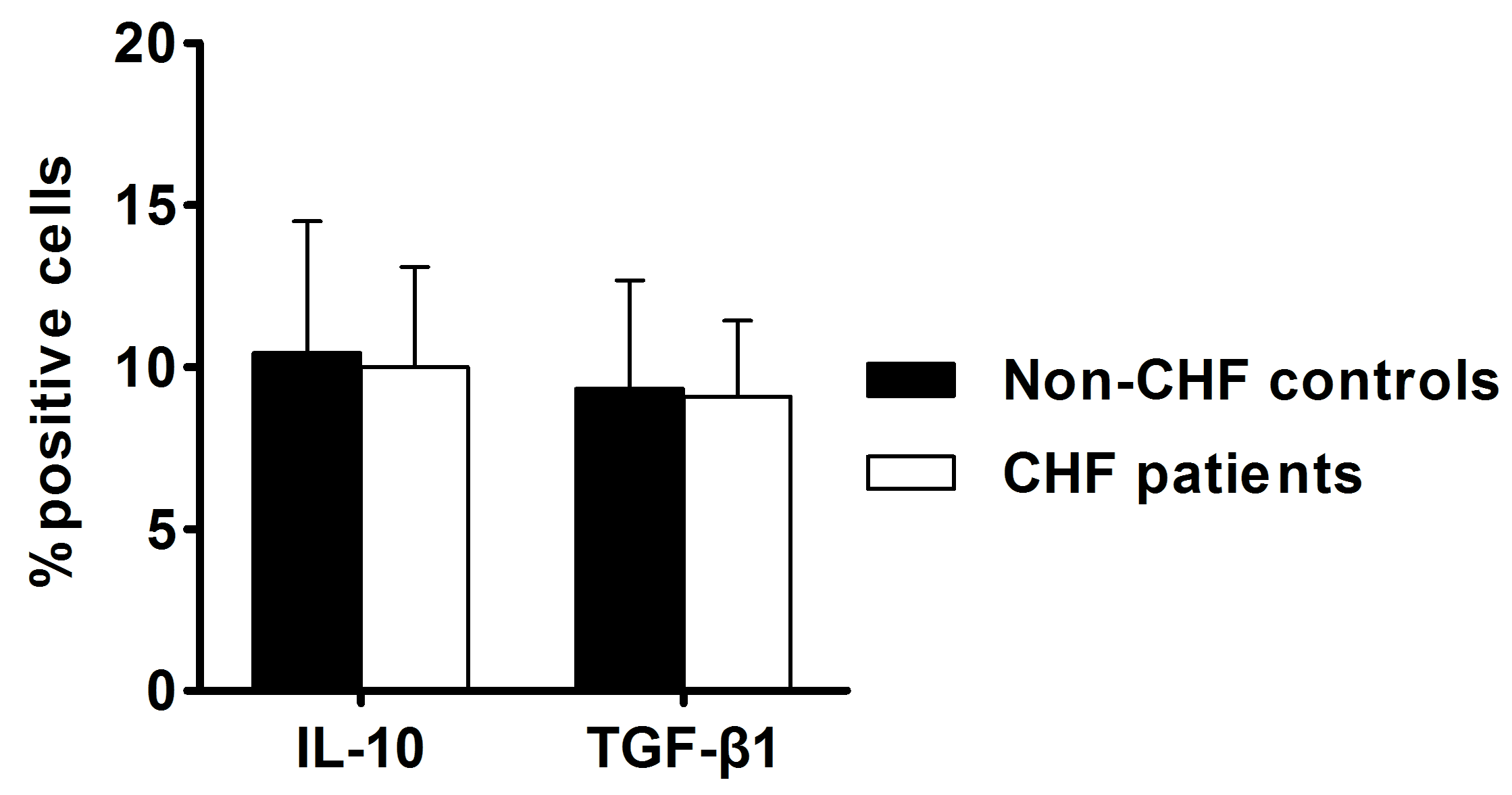

Supplement: Figure S2 — Comparison of intracellular IL-10 and TGF-β1 in CD4+CD25+CD127low Treg between CHF patients (n = 10) and healthy controls (n = 10). (TIF) [file pone.0024272.s002.tif]
